# 171. Gram-Positive Blood Culture Contamination Rates: A Comparative Assessment Between Verigene® and Conventional Method

**DOI:** 10.1093/ofid/ofad500.244

**Published:** 2023-11-27

**Authors:** Nouf K Almaghlouth, Reena Motwani, Roberto Guevara, Umara Khalique, Hebah Alrahamneh, Mohamed Attia, Osaid A Alrahamneh, Mohamed Allokka, Zaid Alrahamneh, Arwa Y Alfarargy, Felix Anyiam

**Affiliations:** Mountain View Regional Medical Center, Las Cruces, New Mexico; UNC Health Southeastern, Lumberton, North Carolina; The Hospital of Providence Transmountain, El Paso, Texas; Jefferson Health, New Jersey, New Jersey; Mountain View Regional Medical Center, Las Cruces, New Mexico; Department of Emergency Medicine, Kasr Alainy University Hospitals, Cairo, Al Qahirah, Egypt; King Hussain Cancer Center, Amman, 'Amman, Jordan; Egyptian Ministry of Health, Mansoura, Ad Daqahliyah, Egypt; Albalqa Applied University, Al Hussain Hospital, Alsalt, Alsalt, Al Balqa', Jordan; Egyptian Ministry of Health, Mansoura, Ad Daqahliyah, Egypt; Centre for Health and Development (CHD),, Port Harcourt, Rivers, Nigeria

## Abstract

**Background:**

The estimated cost of treatment for septicemia among hospitalized patients is $15.4 billion. Approximately 20%-50% of all positive blood cultures were likely false positives. The rates of the reported contaminations in the literature range from 0.6%-12.5%. The false positive report of bacteremia has significantly impacted overall healthcare by increasing mortality rates, prolonging hospital stays, and expanding the cost of care. The use of Verigene® offers polymerase chain reaction (PCR) amplification in < 5 minutes. In our study, we examine the Verigene®, compared to the standard blood culture by the Vitek-2®, to assess for detecting Gram-positive blood contamination (GPBC).

**Methods:**

Blood cultures were collected from patients with sepsis in four hospitals in El Paso, Texas (1/1/2018-5/30/2022). Samples were run through Verigene®. Simultaneously, all specimens were cultured using the conventional method and then incubated for five days. Gram stain used for positive samples. Speciation and antimicrobial susceptibility using Vitek-2®. GPBC in Vitek-2® was identified when one set of two grows microorganisms, universally known as skin flora, without clinical sepsis within 48hr. GPBC in Verigene® was identified when it recognized any skin flora without clinical sepsis within 24hr. The analysis was made using McNemar’s Chi-square test. An observation was statistically significant if the *p-value* was *≤0.05*.

**Results:**

A total of 816 Gram-positive results were obtained. The prevalence of GPBC based on the traditional technique was 98.65% and 64.25% based on the Verigene®. Staphylococcus *epidermidis* was the most detected at 52%, followed by Staphylococcus *aureus* at 26%. The sensitivity of the Verigene® for GPBC detection was 64.22%, and the specificity was 33.33%.

**Figure 1.**
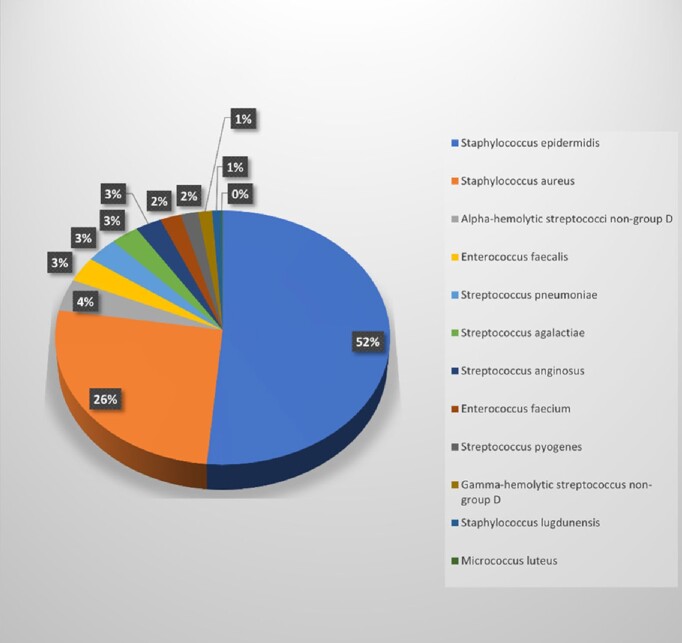
Frequency of Gram-positive Microorganisms Detection

**Figure 2.**
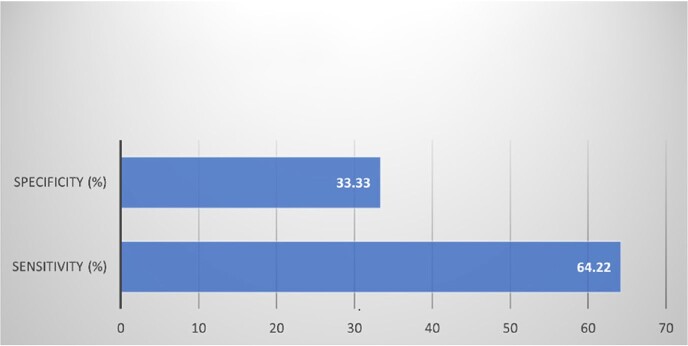
Sensitivity and Specificity of Verigene® in Identification of Contamination in Gram-positive Blood Culture

**Table 1.**
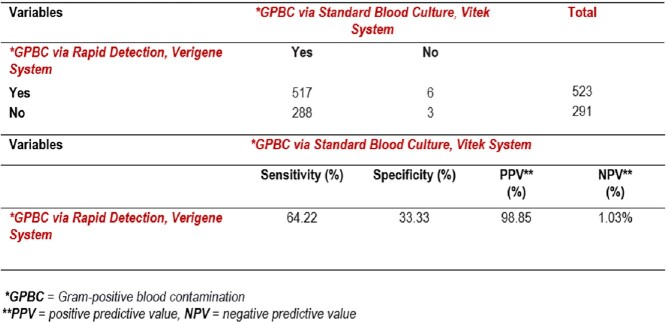
Sensitivity and Specificity of Verigene® in Identification of Contamination in Gram-positive Blood Culture

**Conclusion:**

The use of Verigene® alone in identifying GPBC is insufficient. Despite the higher sensitivity in detecting microorganisms compared to Vitek-2®, this should be paired with clinical judgment and conventional methods. The ability to recognize GPBC by Verigene® was affected by the lack of clinical evidence to exclude sepsis in the first 24hr. This study is the first to evaluate Verigene® for identifying GPBC. Further interventions should be introduced, i.e., reporting titer by PCR on any detection.

**Disclosures:**

**All Authors**: No reported disclosures

